# Why would associations between cardiometabolic risk factors and depressive symptoms be linear?

**DOI:** 10.1186/s12916-014-0199-x

**Published:** 2014-10-28

**Authors:** Peter de Jonge, Annelieke M Roest

**Affiliations:** Interdisciplinary Center Psychopathology and Emotion Regulation (ICPE), University Medical Center Groningen (UMCG), University of Groningen, Postal address: PO Box 30.001, Groningen, 9700 RB the Netherlands

**Keywords:** Cardiovascular disease, Depression, Non-linear, J-shaped curve, Dynamic systems

## Abstract

In medical science, researchers mostly use the linear model to determine associations among variables, while in reality many associations are likely to be non-linear. Recent advances have shown that associations may be regarded as parts of complex, dynamic systems for which the linear model does not yield valid results. Using as an example the interdepencies between organisms in a small ecosystem, we present the work of Sugihara *et al*. in *Science* 2012, **338:**496–500 who developed an alternative non-parametric method to determine the true associations among variables in a complex dynamic system. In this context, we discuss the work of Jani *et al*. recently published in *BMC Cardiovascular Disorders,* (personal communication is incorrect; we never communicated) describing a non-linear, J-shaped curve between a series of cardiometabolic risk factors and depression. Although the exact meaning of these findings may not yet be clear, they represent a first step in a different way of thinking about the relationships among medical variables, namely going beyond the linear model.

Please see related article: http://www.biomedcentral.com/1471-2261/14/139

## Background

Medical science is all about associations. Science becomes relevant if we can predict a variable *y* in which we are interested, from a variable *x* that we have at hand. We aim to develop models capable of making the most accurate predictions, or in other words, to develop the best representations of reality. Modern medical research is centered on associations, with the particular aim to assess whether these associations are causal. Many forms of associations are possible and linear associations are only a particular case. Linearity of an association is, in fact, a rather strong assumption to be made and most associations are likely to be non-linear. Yet, the majority of statistical tests used in medical research are based on that assumption.

### Dynamic systems

In 2012, Sugihara *et al*. published a paper on how causality may be detected in complex ecosystems [1]. Take as an example a pond. In the pond, there is a variety of animal life, including daphnia (’water fleas’), small prey fish and larger predator fish. The pond is an ecosystem in which the presence of several organisms is causally related: an increase in the number of daphnia may be followed by an increase in the number of small prey fish as their food potential has increased. This increase of small prey fish may be followed by an increase in predator fish, effectively reducing the number of small prey fish. By that time, the number of daphnia may have decreased as well, or further increased as a function of the reduction in small fish. These associations are not linear: the association between two variables depends on the magnitude of the two variables and the presence of other internal (in the example, the daphnia and prey fish under the influence of the presence of predator fish) and external variables (for example, water temperature). In this system, the numbers of daphnia and small fish may be positively correlated at some point in time but negatively correlated or not correlated at all at other time points. In other words, in a complex, dynamic system such as a pond, associations between causally linked variables are *dynamic*, and summarizing them in a single value based on a linear function would give an inadequate representation of the true relationships.

### Linear and non-linear associations in medical research

In medical research, we are interested in associations between variables describing the functions of human organisms. The ways associations have been studied are notably different for ecosystems and human organisms. Medical research has mostly been based on small numbers of assessments of many individuals, while ecosystems are often studied as a single entity with many assessments over time. Nevertheless, it is likely that human organisms behave in rather similar ways as ecosystems: there are many interdependent variables, and the association between two of these variables may depend on the magnitude of these variables and the levels of external variables. Take as an example the association between physical activity and depressive symptoms. The linear model would assume that any association between the two would be independent of the magnitude of the two variables (that is, a certain unit of increase in physical activity would lead to a certain increase in mood). However, the reality would probably be that an increase in mood would be achieved particularly when a person does not exercise regularly and would start exercising. However, when someone already exhibits high levels of regular exercise, a further increase in activity level would probably not lead to much mood gains. In addition, the relationship between physical activity and mood is probably bidirectional, meaning that mood could influence physical activity as well. To further complicate matters, the associations between exercise and mood may, in part, depend on external variables too, for example season of the year. As both mood and exercise are probably variables that tend to normalize, there may be complex feedback loops over time that can only be modelled using non-linear techniques applied to repeated assessments of both variables.

### The link between depression and cardiometabolic disease

It is well established that an association between depression and cardiometabolic diseases exists, and that this association is complex and bidirectional [2,3]. The work by Jani *et al.*, published in *BMC Cardiovascular Disorders*, is one of the first steps in attempting to clarify the nature of these associations [4]. In a large sample of individuals with cardiometabolic disease, Jani *et al*. examined the association between blood pressure, body mass index (BMI), cholesterol, and HbA1c and elevated depressive symptoms (that is, a score >7 on the depression subscale of the Hospital Anxiety and Depression Scale (HADS)). All associations were significant and non-linear; more specifically, they were J-shaped. This is remarkable as many of the cardiometabolic risk factors also have J-shaped associations with cardiovascular disease and mortality, for example as shown in [5-7]. While the meaning of these J-shaped associations are unclear at the moment, Jani *et al*. draw a pragmatic conclusion from their data: if the association between cardiometabolic risk factors and depression is J-shaped in this high-risk population, perhaps there is most use in depression screening in persons with either low or high cardiometabolic risk scores (if there is any use in screening).

## Conclusions

Jani *et al*. took an important intellectual step by deviating from the linear model while examining the association between cardiometabolic risk factors and depression. On a conceptual level it may be concluded that we should dedicate more effort into adequate estimation of the shape of associations, instead of only testing the significance of a linear one. We hope that future research will continue on this road and look at these types of associations from a wider perspective, optimizing the shape of estimated associations, while increasingly taking into account potential bidirectional effects and external influences. Hopefully, this will ultimately lead to a better representation of the complexity of reality.
